# Liquefaction and Solidification of Gases

**Published:** 1845-06

**Authors:** 


*Liquefaction and Solidfiication of Gases.*—Dr. Faraday brought forward this subject at the Royal Institution, as the Friday Evening Lecture, Jan. 31.

So long ago as the year 1823, he had been engaged in an endeavor to reduce gases into a fluid state; the means he employed had been to generate the gases in a confined space, and the result had been that he succeeded with the nine following:—Chlorine, hydrochloric acid, sulphurous acid, sulphuretted hydrogen, carbonic acid, euchlorine, nitrous oxide, cyanogen, and ammonia. Since that period the subject has been pursued by others, and a variety of means have been devised to reduce gases to the state of fluid, and further, to solidify some of them. Thus cyanogen and carbonic acid have been rendered solid, and arseniuretted hydrogen, fluid. The means of rendering carbonic acid solid, which may be regarded as the type of the others, as is well known, was devised by Thilorier; and by availing himself of the great cold produced by solid carbonic acid when mixed with ether, together with the air-pump to render the evaporation more rapid, Dr. Faraday has succeeded in reducing to a fluid state the six following gases:—Olefiant gas, phosphuretted hydrogen, hydriodic acid, hydrobromic acid, fluoboron, and fluosillicon; and of rendering solid, sulphurous acid, sulphuretted hydrogen, euchlorine, nitrous oxide, hydriodic acid, and hydrobromic acid.

The method of proceeding, and the principles upon which it is based, are as follow:—The freezing point of mercury is —40°, of carbonic acid,—70°; the cold produced by mixing solid carbonic acid and ether,—105°. By placing the latter under the air-pump the temperature may be reduced to—160°; and Dr. Faraday conceives that by using solid nitrous oxide, instead of carbonic acid, a temperature far lower may be obtained, and by its means, combined with a very high degree of pressure, he thinks oxygen, hydrogen and nitrogen, will certainly be reduced to the fluid or solid state. In fact, he had rendered oxygen fluid, but the enormous pressure had caused the gas to escape through the substance of the metallic stoppers used to close the glass tubes in which the gas was confined. Dr. Faraday had found that common flint glass was quite inadequate to the purpose, and had substituted green bottle glass for it, with perfect success. Tubes made of this glass will bear enormous pressure, and if they give way they do not fly into pieces, but rend open in a vertical direction, so as to do no mischief. By connecting a reservoir of gas with a long metallic tube, at the end of which is attached a bent tube of glass, passing into a bath of solid carbonic acid and ether, under the receiver of an air-pump, and by means of a force-pump pressing into this tube the gas with a force equal to about forty or fifty atmospheres, it becomes fluid or solid in the glass tube, which may then be removed and sealed. It is thus, by combining great pressure with the lowest possible degree of cold, that gaseous bodies are reduced to a solid or liquid state; and Dr. Faraday hopes ere long to decide in this way the disputed questions as to the metallic or non-metallic characters of hydrogen and nitrogen.

Some of the results already obtained are highly curious and important in a scientific point of view; thus ammonia in a solid form is a crystalline white substance, heavier than liquid ammonia, and almost inodorous. Oxide of chlorine is a beautiful orange-red crystalline substance. Alcohol becomes thick and viscid, like cold oil, but does not crystallize; and so with caoutchene, oil of turpentine, &c.—*Lancet.*

